# Hybrid Digital Workflow for Accurate Distal Extension Reproduction in Free-End Removable Dental Prosthesis: A Technical Report

**DOI:** 10.3390/dj14030179

**Published:** 2026-03-17

**Authors:** Thais Marques Simek Vega Gonçalves, Zuila Maria Lobato Wanghon, Liliane da Rocha Bonatto Drummond, Laura Costa Beber Copetti, Renata Blummer, Gabriella Aparecida Cruz dos Reis, Patrícia Pauletto, Analucia Gebler Phillippi

**Affiliations:** 1 Department of Dentistry, Federal University of Santa Catarina (UFSC), Florianópolis 88040-900, SC, Brazil; thais.goncalves@ufsc.br (T.M.S.V.G.); laura.cbc@posgrad.ufsc.br (L.C.B.C.); gabriella.reis@grad.ufsc.br (G.A.C.d.R.);; 2 PPR Digital Private Lab, Piracicaba 13635-900, SP, Brazil; 3 School of Dentistry, Universidad De Las Américas (UDLA), Quito 170517, Ecuador

**Keywords:** removable partial denture, digital workflow, digital design, distal extension, removable prosthesis, metal framework

## Abstract

Background/Objectives: This technical report introduces an innovative hybrid digital workflow that integrates diagnostic plaster-cast scanning with intraoral scanning to produce an accurate 3D-printed model for fabricating distal-extension removable dental prostheses (RDPs). Methods: The technique aims to overcome the challenges of reproducing the mobile mucosa in free-end saddles, a critical factor for denture base accuracy and stability. The workflow began with conventional clinical procedures, including clinical examination, impression-making, and cast surveying. After performing the required mouth preparations according to the prosthetic design, the diagnostic cast was digitized and selectively modified to allow intraoral rescanning. The prepared teeth were then scanned intraorally and merged with the digitalized cast, producing a refined virtual model for CAD-based metal framework design. The framework was digitally designed, 3D-printed to verify adaptation, and cast in cobalt–chromium. Standard RDP fabrication steps were followed, including intraoral framework try-in, fabrication of acrylic bases, occlusal registration, tooth arrangement, and functional and esthetic try-in. The final prosthesis was installed and adjusted without the need for an additional impression. Results: This hybrid workflow enabled a highly accurate reproduction of the distal extension region, outperforming models derived solely from direct intraoral scanning. By digitally capturing the physiological morphology of the mobile mucosa, the method eliminates the need for the traditional altered-cast technique, reducing clinical time, technical sensitivity, and material costs. Conclusions: The proposed approach enhances denture base accuracy, improves adaptation, and promotes more uniform occlusal load distribution in free-end RDPs. This streamlined and reproducible digital protocol offers a clinically relevant advancement, with potential to improve prosthesis stability and long-term outcomes.

## 1. Introduction

Removable dental prostheses (RDPs) remain a widely used treatment modality for partially edentulous patients; however, the conventional fabrication of metallic frameworks is often complex, time-consuming, costly, and susceptible to human error [1,2,3]. Recent advances in computer-aided design and computer-aided manufacturing (CAD-CAM) have transformed RDP fabrication, allowing for digital cast generation, virtual surveying, undercut elimination, and the design and production of 3D-printed or castable frameworks [4]. Recent studies [4,5,6] have shown that digitally fabricated RDPs exhibit comparable, and often superior, framework adaptation compared with conventional methods—particularly in framework seating and posterior strap fit. Additionally, digital design can be completed in approximately 15 min, reducing production time by 60 to 70% [7].

**Figure 1:**
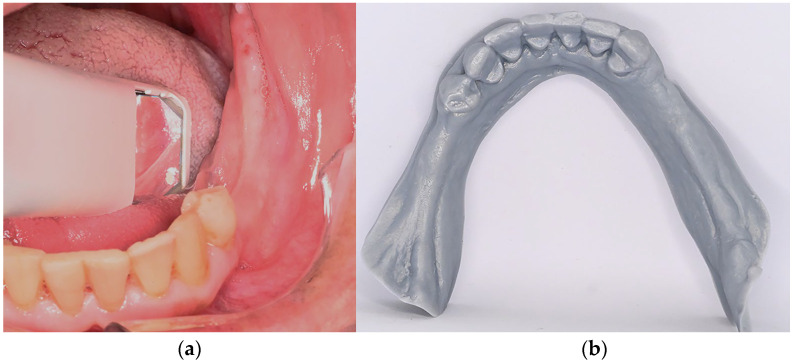
Challenges associated with direct intraoral scanning in posterior edentulous areas. (**a**) Direct intraoral scanning of movable mucosa and the anatomic complexity of distal extension areas can hinder accurate data capture. (**b**) Printed model resulted from direct scanning with insufficient reproduction of the distal extension areas.

Despite these benefits, challenges remain in specific clinical scenarios, such as free-end RDPs. The anatomical complexity and mucosa flexibility of distal extension areas present challenges for direct intraoral scanning, frequently resulting in incomplete data acquisition and insufficient reproduction of these regions in the 3D-printed cast (Figure 1) [7,8]. Because an optimal resin base extension is critical for achieving maximum residual ridge support, inadequate coverage can lead to uneven occlusal load distribution, potential overloading of abutment teeth, and accelerated bone resorption of the alveolar ridge [4,6,8,9]. In such cases, the altered-cast technique has been frequently used to improve denture base adaptation by creating a functional impression of the edentulous region during framework try-in [10,11]. However, this technique is time-consuming and technique-sensitive as it requires additional clinical and laboratory steps, carries a high risk of distortion during cast sectioning and reassembly, and offers limited reproducibility [10,11]. These limitations often increase overall treatment complexity and may compromise the consistency of the final prosthesis [10,11].

In this context, continuing refinement of digital impression techniques in free-end RDPs is crucial to improve functional replication of soft-tissue dynamics and clinical performance of such prostheses, simplifying the process and improving laboratory and clinical workflows [12,13]. Thus, this technical report presents an innovative digital workflow cast impression that integrates the diagnostic plaster-cast scan with the intraoral scan to produce a final 3D-printed cast with accurate reproduction of the distal extensions in free-end removable RDPs.

## 2. Materials and Methods

**Figure 2:**
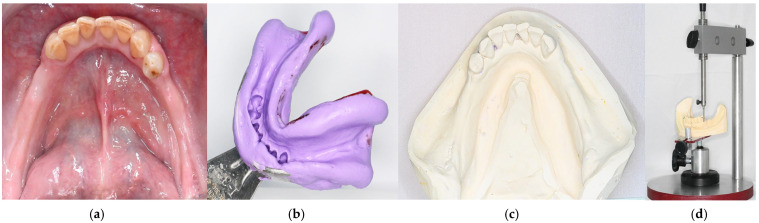
Initial sequence of procedures. (**a**) Intraoral aspect of the initial condition of the lower jaw. (**b**) Conventional impression using a customized metallic tray, border modeling with utility wax, and high-viscosity alginate (Cavex Cream, Cavex, Haarlem, The Netherlands). (**c**) Diagnostic plaster cast of the mandible. (**d**) Surveying of the diagnostic plaster cast to plan mouth preparations.

**Figure 3:**
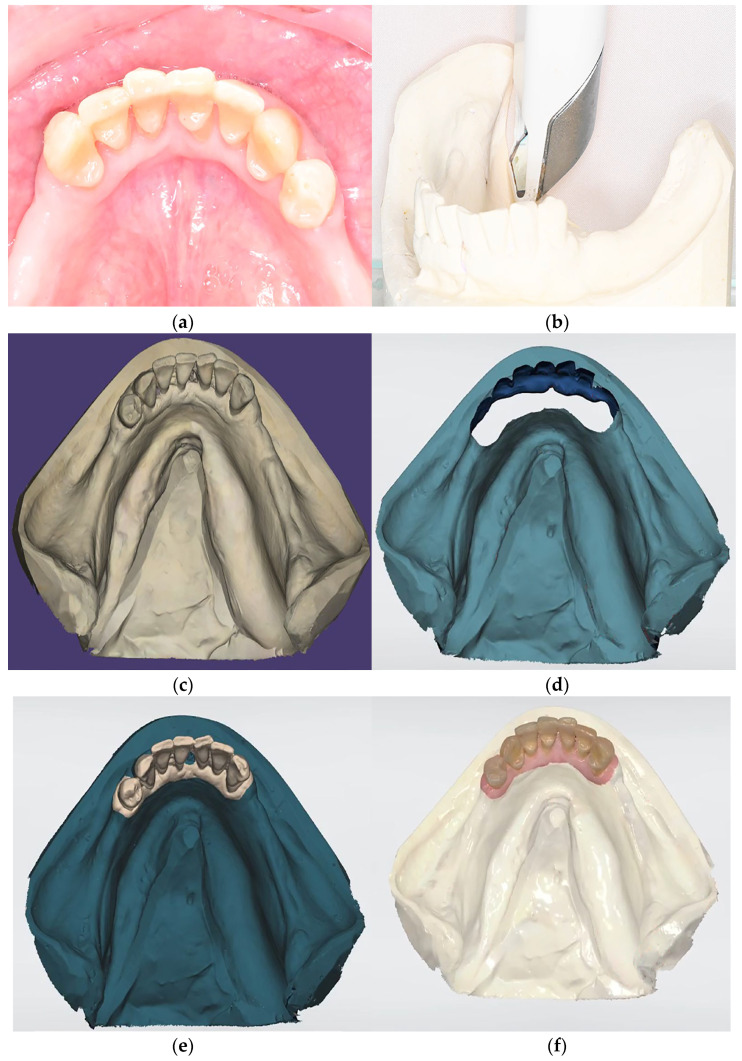
Digital scanning procedures. (**a**) Occlusal view of the prepared teeth, including incisal reconstructions, lingual and occlusal rests, and resin increments on the buccal surface of teeth 34 and 43 to enhance clasp retention. (**b**) Digital scanning of the diagnostic plaster cast. (**c**) Digital image of the diagnostic plaster cast. (**d**) Prepared digital image of the diagnostic plaster cast with block-outs of all areas not related to the prepared teeth. (**e**) Digital image of the diagnostic cast with the regions corresponding to the prepared teeth removed. (**f**) Digital image of the final cast after merging the previous image with the intraoral scanning of the prepared teeth.

**Figure 4:**
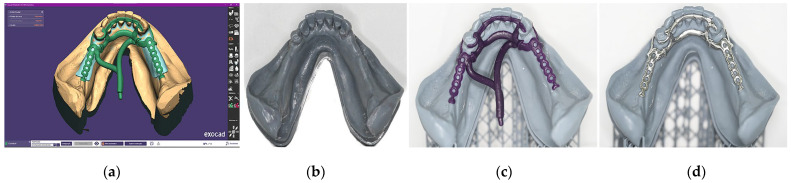
Clinical and laboratory workflow for the fabrication of the mandibular removable dental prosthesis (RDP) and maxillary complete denture. (**a**) Digital design of the metal framework using dental CAD software (Exocad Dental DB, v. 3.1, Exocad, Darmstadt, Germany). (**b**) 3D-printed model (Model Resin Standard, Monile Lab, Curitiba, Brazil). (**c**) 3D-printed resin framework pattern (VisiJet M3 Dentcast, 3D Systems, Rock Hill, SC, USA) to check for fit on the printed 3D model. (**d**) Metallic framework (Co–Cr) adapted to the printed cast.

**Figure 5:**
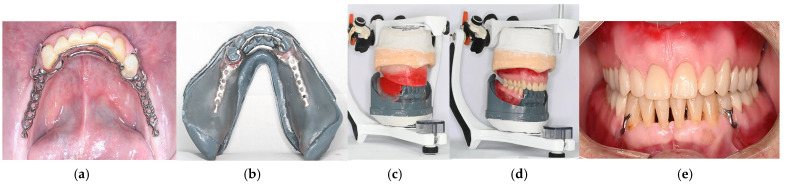
Clinical procedures of RPD manufacturing. (**a**) Framework try-in. (**b**) Bilateral acrylic bases fabricated using auto-polymerized polymethyl methacrylate. (**c**) Final casts mounted on a semi-adjusted articulator. (**d**) Artificial teeth mounted for try-in. (**e**) Intraoral appearance of prosthesis try-in.

**Figure 6:**
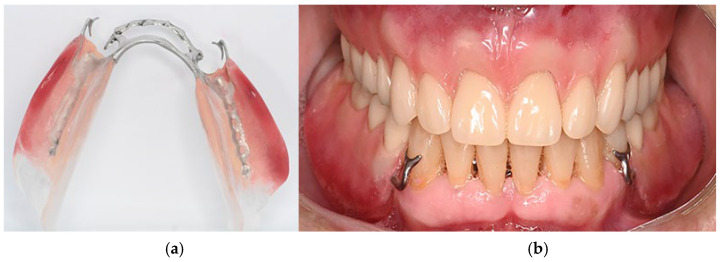
Final aspect of the mandibular RDP. (**a**) Direct view of the acrylic base showing the correct extension of the base (**b**) Clinical aspect of the prostheses in position.

1. Evaluate the clinical appearance of the mandible arch. The mucosa was healthy, and bilateral posterior edentulism was noted, indicating the need for a distal extension RDP (Figure 2a).
2. Make a conventional impression using a metallic stock tray with the border modeling customized with utility wax and a high-viscosity alginate (Cavex Cream; Cavex, Haarlem, The Netherlands) (Figure 2b). The impression was then poured in type IV dental stone (Zero Stone; Dentona, Dortmund, Germany) to obtain a diagnostic cast (Figure 2c). This cast was analyzed using a conventional surveyor to evaluate and plan the necessary mouth preparations for support, stability, and retention of the RDP (Figure 2d).

3. Perform all necessary mouth preparations according to the treatment plan. In this case, incisal reconstructions with direct restorations were completed on all incisors and canines, followed by conventional free-hand lingual rest preparations. Additional composite resin was applied to the buccal surfaces of teeth 34 and 43 to improve clasp retention, and a conventional mesial occlusal rest was prepared on the mesial area of the occlusal face of tooth 34 (Figure 3a).
4. Digitally scan the diagnostic cast using a manual scanner (TRIOS 3; 3Shape A/S, Copenhagen, Denmark) to capture the entire basal area, including the retromolar pads and retromylohyoid fossae (Figure 3b). The scan should be performed in the pre-preparation mode using the CAD software (3Shape Dental System v. 2023.1; 3Shape, Copenhagen, Denmark) to allow subsequent image merging (Figure 3c). For a better performance of the scanner, in the tools button, choose the scan model. Save the file in standard tessellation language (STL) format.
5. In the digitized STL file, apply block-outs to all areas unrelated to the prepared teeth (Figure 3d). Then, remove the regions corresponding to the prepared teeth to enable subsequent intraoral rescanning of these areas (Figure 3e).
6. Switch to the main scanning mode and select “Intraoral Scanning” from the tool’s menu. Perform a direct scan of the prepared teeth using the TRIOS3 (3Shape, 3Shape A/S, Copenhagen, Denmark). The software will automatically merge the intraoral scan with the previously digitized cast to generate the final digital model (Figure 3f). Save the model in STL format and send it to the dental technician for metal framework design and fabrication.

7. The framework was digitally designed by the dental technician using CAD software (Exocad Dental DB, v. 3.1, Exocad, Darmstadt, Germany) (Figure 4a). The connectors, rests, and clasps were modeled according to standard design principles. A 3D model was then printed (Model Resin Standard, Monile Lab, Curitiba, Brazil) to verify the framework adaptation (Figure 4b).
8. A resin framework pattern (VisiJet M3 Dentcast, 3D Systems, Rock Hill, SC, USA) was printed by the dental technician using a 3D printer machine (ProJet MJP 3600 Dental, 3D Systems, Rock Hill, SC, USA), and its adaptation was verified on the printed model (Figure 4c). Once confirmed, the resin pattern was invested and cast in a cobalt–chromium (Co–Cr) alloy following a conventional workflow to produce the definitive metallic framework, which was subsequently rechecked for adaptation on the 3D-printed cast (Figure 4d).

9. Following standard RDP fabrication protocols, make the intraoral try-in of the framework to confirm proper adaptation (Figure 5a). Fabricate bilateral acrylic bases using auto-polymerized polymethyl methacrylate to preserve accurate denture base extension (Figure 5b). Mount the maxillary definitive cast on a semi-adjustable articulator using a facebow. Define the interocclusal registration at centric relation to establish the occlusal vertical dimension in the mandibular wax rim and mount the mandible 3D-printed cast on the semi-adjustable articulator (Figure 5c). After the artificial teeth are mounted (Figure 5d), make the intraoral try-in to evaluate functional and esthetic aspects and obtain patient approval (Figure 5e).
10. Install the polymerized and finished prosthesis and perform the necessary occlusal and functional adjustments (Figure 6).

## 3. Results

**Figure 7:**
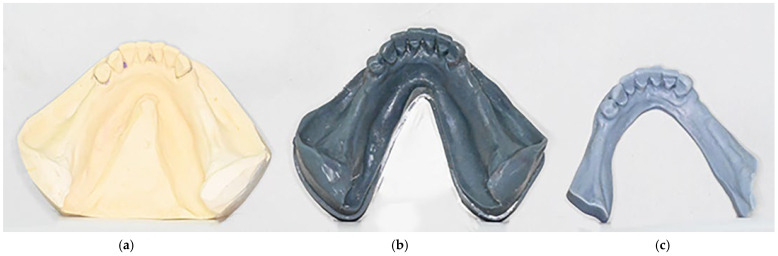
Final aspect of the 3D-printed model obtained from the proposed technique in comparison to direct intraoral scanning. (**a**) Diagnostic cast. (**b**) 3D-printed cast made with the proposed mixed digital workflow. (**c**) 3D-printed cast made with direct intraoral scanning.

The proposed technique resulted in an RDP with an accurately reproduced distal extension of the acrylic base (Figure 6a). Compared to the 3D-printed model generated from direct intraoral scanning, the 3D-printed model produced through the proposed digital workflow showed markedly superior reproduction of the distal extension area (Figure 7). Additionally, in case of thin and/or flaccid mucosa, this correct extension of the resin base allows for pressure equalization throughout the direct impression with light silicon during prosthesis try-in and posterior resin polymerization. These outcomes highlight the technique’s ability to enhance anatomical fidelity in the distal extension region, improving denture base accuracy and overall prosthesis quality.

## 4. Discussion

The described technique introduces an innovative digital workflow for fabricating free-end RDPs, integrating diagnostic cast data with intraoral scans to produce a 3D-printed cast that accurately replicates the distal extension areas. Notably, this method does not modify the conventional sequence, as the diagnostic cast remains part of the routine treatment planning and surveying [1,13].

Accurate reproduction of the distal extension area is critical in Kennedy Class I RDPs to ensure proper support, load distribution, and tissue adaptation, all of which directly affect prosthesis retention, stability, and patient comfort during function [3,9]. Insufficient coverage of the distal basal seat may lead to uneven load distribution, mucosal trauma, food impaction, and prosthesis instability, ultimately overloading abutment teeth and contributing to periodontal deterioration and progressive loss of support [3,8]. However, direct intraoral scanning of distal extension regions remains challenging due to anatomical and physiological constraints, especially in the mandible [7,12]. Tongue interference, mobile soft tissues, and complex mucosal contours often compromise scan accuracy, reducing cast precision and prosthesis adaptation [12,13]. Scan quality is also operator-dependent and may be affected by patient discomfort and data stitching errors [14,15,16].

To address these limitations, clinicians commonly apply the altered-cast technique, which records the edentulous ridge under functional conditions with the framework in place and incorporates this record by sectioning and reassembling the master cast [10,11,12]. Although effective, this method is technique-sensitive, time-consuming, and cost-intensive, requiring additional clinical appointments, materials, and laboratory procedures [11,12]. Cast sectioning and reassembling also introduce the inherent risks of distortion and misalignment, and outcomes depend heavily on clinical and laboratory expertise, reducing reproducibility in routine practice [10,11,12].

The proposed workflow mitigates the limitations of direct intraoral scanning while reducing reliance on altered-cast impressions. By combining intraoral scans of prepared teeth with digitalized diagnostic casts, it enables reliable distal extension reproduction without applying cast sectioning or functional impression procedures [5,15]. The workflow functions as a digital analog of the altered-cast concept instead of a direct replacement, since distal extension support is inferred from the diagnostic cast rather than functionally recorded under controlled loading. Nevertheless, compared with altered-cast-based digital adaptations [14,15,16], the hybrid approach offers greater procedural simplicity and lower technique sensitivity, while maintaining the conventional clinical sequence. In situations involving thin mucosa or pronounced retentive areas, pressure equalization can be achieved using a light-body silicone impression of the edentulous area and consequent inclusion and resin polymerization, without sectioning the master cast or sealing the borders [4,6]. This further reduces laboratory steps and minimizes the risks of distortion and misalignment when compared to the conventional altered-cast method [11].

Nevertheless, the proposed workflow introduces additional digital steps, including scanning, segmentation, and file merging. Therefore, its efficiency advantages should be interpreted primarily as reduced procedural complexity rather than guaranteed reductions in chairside or laboratory time and should be confirmed in future time-controlled studies [17]. Although demonstrated with laboratory-grade scanners and high-resolution printers, the protocol is device-independent and can be implemented with validated desktop scanners and SLA/DLP printing systems of adequate resolution, supporting broader adoption [13]. Economically, the approach shifts costs from consumable materials and labor-intensive laboratory steps toward digital infrastructure [5]. While initial investment in hardware and software may be substantial, marginal per-case costs may decrease in practices with established digital workflows and repeated clinical use [17].

Additionally, this technical workflow provides a clinical illustration rather than quantitative outcome data. Accuracy remains device- and operator-dependent, requiring proper calibration and technical expertise [14,15,16,17]. Moreover, potential alignment errors may introduce subtle inaccuracies, and the need for multi-scan acquisition and large file handling increases data processing and management demands [13]. Thus, well-designed prospective clinical trials are needed to evaluate prosthesis fit, tissue adaptation, functional performance, and patient-centered outcomes before routine clinical implementation can be fully supported.

## 5. Conclusions

The proposed technique integrates intraoral and diagnostic cast scans to digitally reproduce distal extension areas with high accuracy. By preserving the conventional clinical workflow while markedly enhancing distal tissue representation, adaptation, and stability, it offers a practical and accessible advancement in the digital fabrication of RDPs. This approach not only streamlines the process but also elevates the precision and clinical performance of free-end RDPs, offering a practical and accessible solution for digitally fabricated RDPs.

## Data Availability

No new data were created or analyzed in this study. Data sharing is not applicable to this article.

## Abbreviations

The following abbreviations are used in this manuscript:

RDP	Removable dental prosthesis
STL	Standard tessellation language
CAD/CAM	Computer-aided design/computer-aided manufacturing
CAD	Computer-aided design
Co-Cr alloy	Cobalt–chromium alloy
3D	Three-dimensional
